# Evaluation of the Timing of Use of Phosphatidic Acid in the Diet on Growth Performance and Breast Meat Yield in Broilers

**DOI:** 10.3390/ani12243446

**Published:** 2022-12-07

**Authors:** Gregory S. Archer, Eric B. Sobotik

**Affiliations:** Department of Poultry Science, Texas A&M University, College Station, TX 77843, USA

**Keywords:** broiler, phosphatidic acid, mTOR, growth, carcass yield

## Abstract

**Simple Summary:**

Improving feed conversion while increasing growth is a goal of any broiler nutrition program. The use of feed additives to obtain this goal has increased in recent years. Previous research has illustrated that inclusion of phosphatidic acid (PA) in the diet of broiler chickens can increase growth and meat yield. However, it is not known if PA is needed from the start of feeding or if it can be added at later feeding phases to obtain the increased growth and yield.

**Abstract:**

With a growing increase in further processing of poultry, there has been an increased interest in factors, including feed additives, that may improve broiler performance, increase growth, and influence dressing percentage. Mammalian target of rapamycin (mTOR) is known to play vital roles in protein synthesis; mTOR controls the anabolic and catabolic signaling of skeletal muscle mass, resulting in the modulation of muscle hypertrophy. Exogenous phosphatidic acid (PA) can stimulate the mTOR pathway via its activation of the substrate S6 kinase. A study with 648 Cobb 500 male broilers, housed in 36 floor pens (1.11 m^2^) from 1 to 42 days of age was conducted to evaluate the timing of PA (Mediator^®^ 50P, Chemi Nutra, Austin, TX, USA) supplementation on the growth performance and carcass yield of broilers. Dietary treatments included T1, Control (CON), T2, 5 mg/bird/day of PA for 42 days (d0–42, PAA); T3, 5 mg/bird/day of PA for 28 days (d15–42, PAGF); and T4, 5 mg/bird/day of PA for 14 days (d29–42, PAF). All birds were weighed on d14, 28, and 42 to obtain BW (body weight), FCR (feed conversion ratio), and MORT (mortality percentage). On d42, eight birds per pen were processed to determine carcass and breast meat yield. No differences were observed in BW at d14 or d28. At d42, birds fed PAA were heavier (3.73 ± 0.02, *p* < 0.05) than all dietary treatments (3.68 ± 0.02). From d0 to d28, birds fed PAA had the lowest FCR (1.423 ± 0.005, *p* < 0.05) compared to all dietary treatments (1.441 ± 0.005). From d0 to d42, birds fed PAA and PAGF had a lower FCR (1.545 ± 0.014, *p* < 0.05) when compared to the CON (1.609 ± 0.013). No differences were observed in MORT between treatments during growout. Increased BW observed in birds fed PAA translated to increased breast fillet weight (0.772 ± 0.009 kg, *p* < 0.05) when compared to the CON (0.743 ± 0.008 kg). Carcass yields were increased in birds fed PAA (77.48 ± 0.32 kg, *p* < 0.05) when compared to all dietary treatments (76.24 ± 0.16 kg). Utilizing PA for 42 days increased live weights, improved FCR, increased carcass yield, and increased breast fillet weight at processing. Results from this study indicate that supplementation of PA during all phases of growth may increase the production efficiency of broilers.

## 1. Introduction

There have been tremendous improvements in growth rates and breast meat yield of broiler chickens over the last several decades. This has been in response to the increased demand for commercial meat production as worldwide meat consumption is increasing. A large majority of meat consumption worldwide is poultry, as it is typically low priced, highly nutritional, and highly suitable for processing and further processing [1]. Although poultry is generally a very efficient agriculture commodity, the poultry industry is always looking form means to further improve growth, feed conversion, and yield. While this has been carried out via genetics [2] historically, the use of feed additives has also grown, and new products and modes of action are always of interest. Products such as probiotics, prebiotics, minerals, and various other feed additives are commonly used. However, other naturally physiological processes related to growth have not fully been examined. One such processes involves the mTOR pathway which is involved in muscle growth. A substance called phosphatidic acid, which is found in many foodstuffs and is often used in humans to aid in muscle growth [3], has been shown to be key to this pathway.

The increase in muscle mass and muscle growth occurs during development but also in response to mechanical overload or exercise [4]. Often mechanical stimuli play a significant role in the regulation of skeletal muscle mass, and it is determined by the mammalian target of rapamycin (mTOR), a protein kinase [5]. The activation of mTOR signaling is, in fact, sufficient to induce an increase in muscle fiber size [5]. The mTOR pathway is activated by the messenger phosphatidic acid (PA) following mechanical stimulation. During mechanical stimulation, intracellular concentrations of PA increase, activating the mTOR pathway via a phosphoinositide 3-kinase and mitogen-activated protein kinase independent mechanism [5]. It has been demonstrated that exogenous PA can stimulate the mTOR pathway via its activation of the substrate S6 kinase [6]. However, the binding of PA to S6 kinase may occur independently of mTOR [7], suggesting that PA may augment the signaling response when mTOR is activated by exercise. However, previous research [8] demonstrated that exercise was not needed to increase growth in broiler chickens. Sobotik et al. [8] found that simply feeding broilers 5 mg/day of PA throughout the entire rearing period resulted in increased bird weights, feed conversion, and breast weights.

Growth in broiler chickens is well described as a sigmoid curve with an initial exponential development phase, followed by an intermediate or transitory phase, and finally, an inhibitory growth phase which consists of a gradual reduction in the growth rate following an asymptotic increase in body weight [9]. In broilers, posthatch muscle growth through the process of hypertrophy is mediated by the adult myoblast stem cell population (satellite cells). The satellite cells hypertrophy by fusing with and donating their nuclei to existing muscle fibers resulting in muscle growth [10,11]. Phosphatidic acid has been shown to simulate myoblast proliferation through interaction with LPA1 and LPA2 receptors [12]. The first week posthatch in broilers, especially those selected for rapid growth, has been shown to be the period of maximal satellite cell activity and the most important for muscle production [13]. Although overall growth rate has been observed to increase and peak at around 30 days of age and then decrease [14], breast muscle mass has been observed to peak around 40 days of age [14]. Therefore, it is not clear at which time point PA supplementation in the feed should begin or for how long.

The mTOR pathway is connected to growth factor signaling via the insulin/insulin-like signaling system (IIS) [15]. A decrease in IIS/mTOR signaling activity is associated with increased resistance to certain types of stress, which plays a vital role in the adaption to different stress conditions [16,17], which could also aid in broiler performance and growth in addition to its positive correlation with muscle growth. Therefore, IIS and mTOR pathways play an important role in hypertrophic muscle accumulation [18]. PA function is mainly related to the activation of the target of the rapamycin complex 1 (TORC1) signaling pathway [19,20,21], which regulates cell growth and metabolism by sensing the intracellular nutritional status [22,23]. The TORC1 signaling pathway is also related to growth factor signaling via the IIS [15]. PA has also been shown to ameliorate stomach mucosal injury in mice [24], indicating that PA might improve the health of digestive track. The mTOR pathway is also related to immunity and stress susceptibility [16,17,25]. If this is true, it could be another possible route of improved nutrient utilization, resulting in improved growth and efficiency totally separate from the muscle growth pathways. This could make feeding PA throughout the rearing period beneficial beyond the initial satellite cell muscle proliferation growth period.

The objective of this study was to evaluate the effect of the timing of dietary PA supplementation on broiler chicken growth and meat yield. To accomplish this, an experiment was conducted that evaluated adding PA to the diet at different dietary phases. It is hypothesized that PA supplementation will increase muscle growth and yield, and that the length of the inclusion may be a factor.

## 2. Materials and Methods

### 2.1. Animals and Husbandry

A total of 648 male Cobb 500 broilers were used in this experiment. Birds were equally housed at 18 birds per pen across a total of 36 pens (0.91 m × 2.74 m). Birds were randomly assigned to each pen; however, initial pen weights were equalized (0.79 kg). Each pen was lined with used pine shavings equipped with one bell feeder and nipple drinking system. Pens were blocked within and the treatments were assigned at random to one of four dietary treatments: control diet (CON) or control diet with Mediator^®^ Phosphatidic Acid (Mediator^®^ PA, Chemi Nutra, Austin, TX, USA) added at a rate so birds consumed 5 mg/bird/day for all 42 days of the trial (PAA), PA for days 15–42 of the trial (PAGF), or PA for days 29–42 of the trial (PAF). Birds were fed a three-phase diet consisting of a starter (days 0–14, crumble), grower (days 15–28, pellet), and finisher (days 29–42, pellet) phase. All feed was pelleted at 185 °F. Birds were allowed ad libitum access to feed and water. A photoperiod of 24L:0D was maintained to 10 days of age and then reduced to 20L:4D for the remainder of the study. Birds were raised to 42 days of age. The birds were managed according to the guidelines outlined approved by the Texas A&M University animal care and use committee (IACUC 2018-0181).

### 2.2. Growth, Feed Conversion, Processing Parameters

The birds in each pen were weighed at day 0, 14, 28, and 42. Body weight gain was calculated by subtracting day 0 weight from the weight at each weigh day. Feed was weighed before it was added to the feeder in each pen and residual feed was weighed back on weigh days so that feed intake could be calculated. Feed conversion ratio (FCR) was calculated by dividing the total feed intake per pen by the total body weight gain per pen and corrected for mortality (mortality was weighed and added to final pen weights). At the end of each experiment following 8 h of feed withdrawal, 8 birds per pen were processed and deboned for carcass, tender, wing, leg quarter and breast yields. 

### 2.3. Statistical Analysis

All data was analyzed via One-Way ANOVA using the GLM model (SPSS Software 28, IBM, Armonk, NY, USA) with treatment means deemed significantly different at *p* ≤ 0.05. Treatment means that were determined to be significant were further separated using Duncan’s Multiple Range Test.

## 3. Results

The live bird weights are presented in Table 1. No differences were observed in bird weights from d0 to d28 (*p* > 0.05). At d42, the PAA treated birds weighed more (3.725 kg) than those of all other treatments (*p* < 0.05, average 3.679 kg).

The data for feed conversion is presented in Table 2. There was no difference between treatments in FCR during the d0–14 period (*p* > 0.05). The PAA treatment had lower FCR (1.423 and 1.535) than the CON treatment from d0–28 and d0–42 periods (*p* < 0.05, 1.449 and 1.609). The PAGF also had better FCR (1.556) than the CON treatment from d0–42 (*p* < 0.05).

Processing part weight data are presented in Table 3. The PAA (0.772 kg) and PAF (0.735 kg) treatments had heavier breast weights than the CON treatment (*p* < 0.05, 0.743 kg). No other differences were observed in part weights (*p* > 0.05).

The processing yield data is presented in Table 4. The PAA treatment had greater overall carcass yield (77.48%) and lower leg quarter yield (30.44%) than all other treatments (*p* < 0.05, average 76.24% and 31.06%, respectively).

## 4. Discussion

As the demand for meat continues to increase globally and with poultry meat being a large percentage of the meat consumed globally, there is increased need to increase production and efficiency. Factors such as gut health [26] and stress [27] are known to influence broiler growth and efficiency. It has been estimated that at least 85% of improvement to broiler performance [28] is related to genetic selection. While genetics continue to be optimized for increased growth and efficiency at a rate of around 2–3% each year [28], that is only one part of the effort to continue the increase in bird growth. Many feed supplements have been and continue to be investigated to improve growth rates and feed conversion in poultry. These supplements range from products that look to improve gut health and microbiota [29] to those looking to reduce the detrimental effects of stressors [30]. Manipulation of dietary components, such as minerals [31] and ammino acids [32], have also been investigated. Furthermore, methods to increase muscle growth via increasing satellite cell proliferation have also been investigated. However, little research has been undertaken on looking at dietary methods to stimulate the mTOR pathway in broilers, which is involved in satellite cell proliferation, immunity, gut health, and stress susceptibility.

It has been demonstrated previously that exogenous supplied PA can result in stimulation of the mTOR pathway [6,33], which can lead to increased muscle growth. This muscle growth has generally been thought to be related to mechanical overload or exercise [4]. Naturally, when mechanical stimulation occurs, PA is released, stimulating the mTOR pathway; however, supplementing PA exogenously in the feed circumvents this need for mechanical stimulation to produce PA. In fact, previous research [8] demonstrated that, even without exercise, feeding PA to broiler chickens resulted in increased growth, feed conversion, and yield. It was not clear from that study, however, for how long PA needed to be fed to growing chickens to obtain those results.

This current study attempted to investigate that question by feeding PA for the entire growth period (0–42 days of age), starting after the initial growth period (15–28 days of age) or for just the last growth phase of production (29–42 days of age). This current study clearly demonstrated that feeding broiler chickens for only two or four weeks prior to harvesting them did not improve growth, feed conversion or carcass yield. This current study demonstrated that broilers need to be fed from the start of production to obtain the performance improvements observed in Sobotik et al. [8]. The current study saw an overall improvement in body weight at d42 in PAA fed birds and specifically increased breast weight over birds not supplemented with PA or birds with PA supplementation that started after the first 14 days of growth. This may indicate that PA played a critical part in muscle cell growth during satellite cell proliferation seen by others [12]. In broilers, the maximum satellite cell activity occurs in the first week after hatch [13], although the overall growth in broilers increases until about 30 days of age [14]. Environmental factors, such as lighting [34] or heat exposure [35], have been shown to stimulate satellite cell activity in broilers. It has also been shown that increased activity during the first week of life was concluded to result in increased growth later in life. Similar to those studies, PA likely stimulated satellite cell activity in the PAA treatment during the early growth phase, resulting in the increased growth observed by d42 of age.

This current study observed that the PAA treatment had reduced leg quarter weights compared to all the other treatments. Considerable research has been conducted on the growth characteristics of skeletal muscle in broilers [36,37,38,39] and those studies agreed that breast muscle protein synthesis is more rapid in fast-growing broilers than in the slower growing chickens. Furthermore, they concluded that the breast muscle grows faster than leg muscles during the early stages of posthatching development. Therefore, the PA supplementation may have led to increased nutrient utilization for breast muscle growth over leg muscle growth. While growth rate has been observed to increase to peak at around 30 days of age [14], breast muscle mass has been observed to peak at around 40 days of age [14] and, therefore, this may also be a factor in the increased breast meat weights and decreased leg quarter weights.

Overall, feed conversion was also improved in the PAA treatment compared to other treatments, indicating that supplementation in that treatment allowed for more efficient nutrient utilization, though the exact mechanism for that improvement merits further research.

It is possible that while feeding PA at later phases of the broiler diet may still result in stimulation of the mTOR pathway, as it has been used in older humans to increase muscle growth [3], the results of this current study, however, were insufficient to result in significant improvements compared to diets without PA added. As broilers have a sigmoid growth curve [9], the addition of PA to the feed appears to be more important to start from the beginning of production. This is the period where maximal satellite cell activity occurs [13] and PA has been demonstrated to stimulate the proliferation of these cells [12]. It is not clear from this current research whether just the initial starter diet phase is needed, as the feeding of PA either for the entire 6-week growout or for the last two or four weeks were only investigated. Future research is needed to determine if feeding only in the starter phase could result in the increased growth, feed conversion, and yield observed in this study. It could be that the starter phase is a critical period because of the satellite cell proliferation alone or the increased performance could be an additive effect over time, as other factors such as increased gut health and immunity can also improve growth and PA/mTOR has been shown to be involved in both [24,25,33]. Stress susceptibility has also been observed to be influenced by the mTOR pathway [16,17]. All these factors can greatly impact broiler growth and efficiency and could support the need for long-term PA supplementation, not just during the starter phase. This also needs further investigation, as growth rate peaks at approximately 30 days of age and breast muscle growth at 40 days [14].

## 5. Conclusions

This study confirmed that utilizing PA in the diet of broiler chickens can improve body weight, FCR, and carcass yield. However, to obtain all these positive results, they must be fed from the start of the growout, as starting during the grower or finisher phase did not result in improvements. It is unclear whether the same results could be obtained by just feeding during the starter phase and more research is needed to confirm this. However, it is clear that utilizing PA in the feed can increase production efficiency in broiler chickens.

## Figures and Tables

**Table 1:** Average live bird weights (kg) of broilers fed either a control diet or control diet with 5 mg/bird/day of phosphatidic acid added from d0–42 (PAA), d15–42 (PAGF), d29–42 (PAF).

Treatment	Day 0	Day 14	Day 28	Day 42
Control	0.046	0.529	1.799	3.636 ^a^
PAA	0.046	0.527	1.813	3.725 ^b^
PAGF	0.046	0.526	1.798	3.692 ^a^
PAF	0.046	0.526	1.812	3.709 ^a^
Pooled SEM	0.000	0.004	0.007	0.014

^a,b^ Differing superscripts within the column indicate significant differences *p* < 0.05.

**Table 2:** Feed conversion ratio (FCR) of broilers fed either a control diet or control diet with 5 mg/bird/day of phosphatidic acid added from d0–42 (PAA), d15–42 (PAGF), d29–42 (PAF).

Treatment	FCR d0–14	FCR d0–28	FCR d0–42
Control	1.178	1.449 ^a^	1.609 ^a^
PAA	1.161	1.423 ^b^	1.535 ^b^
PAGF	1.160	1.434 ^a^	1.556 ^b^
PAF	1.182	1.442 ^a^	1.576 ^ab^
Pooled SEM	0.008	0.004	0.009

^a,b^ Differing superscripts within the column indicate significant differences *p* < 0.05.

**Table 3:** Processing part averages (kg) of broilers fed either a control diet or control diet with 5 mg/bird/day of phosphatidic acid added from d0–42 (PAA), d15–42 (PAGF), d29–42 (PAF).

Treatment	Carcass Weight	Breast Weight	Tender Weight	Wing Weight	Leg Quarter Weight
Control	2.736	0.743 ^a^	0.131	0.287	0.857
PAA	2.779	0.772 ^b^	0.133	0.295	0.845
PAGF	2.729	0.735 ^a^	0.131	0.286	0.853
PAF	2.743	0.744 ^a^	0.130	0.285	0.860
Pooled SEM	0.022	0.010	0.002	0.005	0.008

^a,b^ Differing superscripts within column indicate significant differences *p* < 0.05.

**Table 4:** Processing yield averages (%) of broilers fed either a control diet or control diet with 5 mg/bird/day of phosphatidic acid added from d0–42 (PAA), d15–42 (PAGF), d29–42 (PAF).

Treatment	Carcass Yield	Breast Yield	Tender Yield	Wing Yield	Leg Quarter Yield
Control	75.88 ^a^	27.18	4.81	10.52	31.39 ^a^
PAA	77.48 ^b^	27.77	4.80	10.61	30.44 ^b^
PAGF	76.18 ^a^	26.93	4.79	10.51	31.31 ^a^
PAF	76.66 ^a^	27.10	4.77	10.41	31.36 ^a^
Pooled SEM	0.31	0.22	0.05	0.17	0.22

^a,b^ Differing superscripts within the column indicate significant differences *p* < 0.05.

## Data Availability

The data presented in this study are available on request from the corresponding author. The data are not publicly available due to privacy.
